# The clinical utility of microarray technologies applied to prenatal cytogenetics in the presence of a normal conventional karyotype: a review of the literature

**DOI:** 10.1002/pd.4209

**Published:** 2013-09-08

**Authors:** Jonathan L A Callaway, Lisa G Shaffer, Lyn S Chitty, Jill A Rosenfeld, John A Crolla

**Affiliations:** 1 Wessex Regional Genetics Laboratory, Salisbury District HospitalSalisbury, UK; 2 Faculty of Medicine, Department of Human Genetics and Genomic Medicine, University of Southampton, Southampton General HospitalSouthampton, UK; 3 Paw Print Genetics, Genetic Veterinary Sciences, Inc.Spokane, WA, USA; 4 Clinical and Molecular Genetics Unit, UCL Institute of Child HealthLondon, UK; 5 Great Ormond Street NHS Foundation TrustLondon, UK; 6 University College London Hospitals NHS Foundation TrustLondon, UK; 7 Signature Genomic Laboratories, PerkinElmer, Inc.Spokane, WA, USA

## Abstract

**ABSTRACT:**

The clinical utility of microarray technologies when used in the context of prenatal diagnosis lies in the technology's ability to detect submicroscopic copy number changes that are associated with clinically significant outcomes. We have carried out a systematic review of the literature to calculate the utility of prenatal microarrays in the presence of a normal conventional karyotype. Amongst 12 362 cases in studies that recruited cases from all prenatal ascertainment groups, 295/12 362 (2.4%) overall were reported to have copy number changes with associated clinical significance (pCNC), 201/3090 (6.5%) when ascertained with an abnormal ultrasound, 50/5108 (1.0%) when ascertained because of increased maternal age and 44/4164 (1.1%) for all other ascertainment groups (e.g. parental anxiety and abnormal serum screening result). When additional prenatal microarray studies are included in which ascertainment was restricted to fetuses with abnormal ultrasound scans, 262/3730 (7.0%) were reported to have pCNCs. © 2013 The Authors. *Prenatal Diagnosis* published by John Wiley & Sons Ltd.

## INTRODUCTION

Over the past few years, microarray analysis, also known as molecular karyotyping or chromosomal microarray analysis, has gradually replaced conventional G-banded karyotyping as the frontline diagnostic test for children and adults presenting with a wide range of neurodevelopmental phenotypes with or without associated congenital abnormalities.^1^–^4^ In many countries, notably in the USA and Europe, the transfer from karyotyping to microarrays in the postnatal constitutional setting is now widespread, but the application of this technology to prenatal cytogenetics has lagged behind the postnatal implementation largely because of the perceived difficulties of interpreting variants of unknown significance (VOUS) in the context of an ongoing antenatal diagnosis. Prenatal microarrays have been more readily adopted in specific situations. For example, the use of microarrays in cases of known chromosomal abnormalities that were initially identified by karyotyping allows for further characterisation of the chromosomal breakpoints and the genes involved. Also, a number of smaller prenatal studies have restricted the comparison of microarrays and conventional karyotyping to fetuses ascertained with ultrasound abnormalities.^5^–^13^

Recently, several large-scale prenatal microarray studies have been published in which the diagnostic yields and the utility of microarrays and conventional karyotyping have been compared. In these studies, prenatal microarrays were used for all categories of prenatal diagnosis ascertainment including abnormal ultrasound scans, maternal age, abnormal serum screening result (with or without associated ultrasound), and parental anxiety as well as a history of chromosome abnormalities.^14^–^17^

In this paper, we have attempted to determine the underlying rate of copy number changes with associated clinical significance (pCNCs) that can be detected by microarrays in the prenatal diagnostic setting but have restricted our inclusion criteria to those cases where a pCNC has been detected in the presence of a normal conventional karyotype. This review therefore excludes the evaluation of cases where normal microarray results may mask a balanced chromosome rearrangement that could have clinical significance, for example apparently balanced translocations or inversions in conjunction with normal microarray profiles. We have presented our results to try and estimate the overall detection rate of prenatal pCNCs within the subclassifications of primary referral criteria.

## METHODS: INCLUSION AND EXCLUSION CRITERIA

The search for suitable papers focused on prenatal studies using microarray technology. The advanced search function of the NCBI database, online resource PubMed (http://www.ncbi.nlm.nih.gov/pubmed/advanced), was used with the word ‘prenatal’ combined with the following terms to describe microarrays: ‘microarray’, ‘array’, ‘chomosomal microarray’, ‘array comparative genomic hybridisation’, ‘cma’, ‘CGH’ and ‘aCGH’. The titles and abstracts of the identified articles were then checked against predetermined criteria for eligibility. Bibliographies of relevant papers were manually interrogated for further papers not identified by electronic searches. Experts in the field were also contacted for completeness of the literature review (Lisa G. Shaffer and Jill A. Rosenfeld).

Our initial review of the literature identified 22 suitable papers^5^–^26^ published between 2005 and 2013, which were then subdivided into those where all categories of prenatal ascertainment were included in the study design^14^–^17^ and those where the analyses were restricted to fetuses presenting with an abnormal ultrasound scan.^5^–^12^ It should be noted that only papers where the microarray results, conventional karyotypes and the final clinical interpretations were all recorded have been included in this review. The strict inclusion criteria of papers also required the ability to identify the number of cases from each ascertainment category with a pCNC in the presence of a normal karyotype, calculate the total number of karyotypically normal cases tested by CMA belonging to each category and correlate specific raw copy number change data supplied by the authors to numbers provided in their summary tables. If it was not possible to elucidate this information from the paper, or obtain it through personal communication with the authors, then the paper was not included in the review. Based on these inclusion criteria, the relatively large data sets from Breman *et al*.,^19^ Park *et al*.^18^ and Armengol *et al*.^20^ were not included in the final tables.

There were a number of difficulties encountered when trying to make direct comparisons between studies including the following: (i) We have used and presented, without further interpretation or comment, the genome coordinates and clinical interpretation of pCNCs as published by the individual groups and have reproduced the original calls and associated interpretations in the Appendices. (ii) The studies included have used a variety of microarray platforms comprising differences in design and resolution (e.g. from ‘targeted’ 1 Mb BAC arrays to custom designed 44k oligo or 125k SNP arrays). It should be noted that we have only included abnormalities involving a change in copy number and have therefore excluded abnormalities such as uniparental disomy, which were detected by SNP arrays. (iii) We made the decision not to include copy number changes that were classified purely as VOUS. It should be noted here that some authors (e.g. Wapner *et al*.^14^) include a number of potentially significant VOUS within their category of copy number changes with clinical significance, and we have included these cases within the ‘pCNC’ group to try and determine the underlying rate of cases deemed to have clinical consequences. VOUS rates frequently depend on both the coverage of the array and the laboratory reporting policy employed, making comparisons across studies difficult to do meaningfully. The stratification of the relative risk associated with any particular VOUS can also determine whether it is considered to fall within a low, moderate or high risk of adverse clinical consequences, and this stratification is reflected in the evidence-based subclassification of VOUS as outlined by the International Standards for Cytogenomic Arrays (https://www.iscaconsortium.org/). In many of the papers reviewed, no such risk stratification was presented with the exception of Wapner *et al*.^14^ who employed an expert review panel to stratify risks associated with VOUS encountered throughout the course of their study. Although the clinical management of VOUS especially within the prenatal setting is important, we felt that the focus of this review should be restricted to those cases that were interpreted and therefore reported to have clinically significant prenatal pCNCs in the presence of a normal karyotype.

## RESULTS

A list of the published results can be found in the online appendix. The inclusion criteria used in this review meant that a number of relatively large-scale studies were not included principally because it was not possible to correlate individual karyotypic and microarray results^18^ and/or to break down results by different ascertainment groups.^19^,^20^ Other studies were not cited individually because their results were included in other large-scale studies (e.g. Kleeman *et al*.,^13^ Maya *et al*.^21^ and Coppinger *et al*.^22^ are included in the study by Shaffer *et al*.^15^).

### Overall prenatal detection rate for pCNC in the presence of a normal conventional karyotype

In Table 1, it can be seen that we have limited our review to four large-scale studies in which the data are broken down into three main ascertainment categories. It should also be noted that because of differing study designs, there is significant variability with respect to the numbers reflected in the ascertainment groups; for example, ∼51% and ∼38% of cases recruited by Wapner *et al*.^14^ and Fiorentino *et al*.,^17^ respectively, were for advanced maternal age compared with only 6% by Shaffer *et al*.^15^ By comparison, the proportion of cases recruited because of an abnormal ultrasound ranged from ∼2.5% by Fiorentino *et al*.^17^ to ∼80% by Shaffer *et al*.^15^ Despite this variation in study design we decided to pool these data, which results in 25% of cases recruited following an abnormal ultrasound scan, 41% with advanced maternal age and 34% for the other ascertainment categories.

**Table 1:** Summary of pCNC microarray findings in routine prenatal diagnosis in the presence of a normal karyotype

Study	Sample size^a^	Platform	Total pCNC (%)	Ascertainment		
				Abnormal ultrasound (%)	Maternal age (%)	Other (%)^b^
Wapner *et al*.^14^	3822	Targeted with 1 Mb backbone	96 (2.5)	45/755 (6.0)	34/1966 (1.7)	17/1101 (1.5)
Shaffer *et al*.^15^	2587	Various	142 (5.5)	131/2081 (6.3)	0/161 (0.0)	11/345 (3.2)
Lee *et al*.^16^	3080	BAC targeted/60k oligo	35 (1.1)	20/180 (11.1)	10/1891 (0.5)	5/1009 (0.5)
Fiorentino *et al*.^17^	2873	BAC targeted	22 (0.8)	5/74 (6.8)	6/1090 (0.6)	11/1709 (0.6)
Total	12 362	Various	295 (2.4)	201/3090 (6.5)	50/5108 (1.0)	44/4164 (1.1)

a Normal conventional karyotypes only.

b Including parental anxiety, history of chromosome abnormality, and abnormal serum screening result.

These pooled data show that in the presence of a normal conventional karyotype, 295/12 362 (2.4%) of cases reported overall were found to have a pCNC compared with 201/3090 (6.5%) following an abnormal ultrasound, 50/5108 (1.0%) when ascertained because of increased maternal age and 44/4164 (1.1%) for all other ascertainment groups.

### Fetuses presenting with ultrasound abnormalities

The results for the eight targeted studies included in these analyses^5^–^12^ are summarised in Table 2. Amongst these targeted studies, the detection rate for pCNCs ranged from 6.1% to 13.3%, but by pooling these data, 61/640 (9.5%) cases were interpreted to have a pCNC. In Table 2, we have also included the abnormal ultrasound abnormality ascertainment category from Wapner *et al*.,^14^ Shaffer *et al*.,^15^ Lee *et al*.^16^ and Fiorentino *et al*.,^17^ from which it can be seen that 262 of the 3730 (7.0%) abnormal ultrasound cases overall were classified as having pCNCs.

**Table 2:** Prenatal microarray studies focused on abnormal ultrasound (AUS) only

	Sample size^a^	Platform	No. pCNC	pCNC %
AUS only studies				
Tyreman *et al*.^5^	106	Affymetrix Gene Chip 6.0	11	10.4
D'Amours *et al*.^6^	49	BAC/105 and 135k oligo	6	12.2
Evangelidou *et al*.^7^	15	1 Mb BAC	2	13.3
Valduga *et al*.^8^	50	44k oligo	5	10.0
Faas *et al*.^9^	30	250k SNP	2	6.7
Srebniak *et al*.^10^	199	105k SNP	16	8.0
Le Caignec *et al*.^11^	49	BAC targeted	3	6.1
Rooryck *et al*.^12^	142	60k oligo	16	11.3
Subtotal	640		61	9.5
Other studies				
Wapner *et al*.^14^	755	Targeted with 1 Mb backbone	45	6.0
Shaffer *et al*.^15^	2081	Various	131	6.3
Lee *et al*.^16^	180	BAC targeted/60k oligo	20	11.1
Fiorentino *et al*.^17^	74	BAC targeted	5	6.8
Subtotal	3090		201	6.5
Combined studies	3730		262	7.0

a Normal conventional karyotypes only.

## DISCUSSION

The primary purpose of this review was to estimate the ‘added’ diagnostic value of microarray technology when applied to cytogenetic prenatal diagnosis especially in those cases where conventional G-banded analysis provides a normal chromosome result. The headline figures are compelling in that from all ascertainment groups comprising 12 362 cases, microarrays revealed 295 (2.4%) of cases with cryptic abnormalities interpreted to have clinical significance to the ongoing pregnancy, and this increases to 7.0% of cases following an abnormal ultrasound scan, and in ∼1% of combined cases with advanced maternal age or other referrals such as parental anxiety, history of chromosome abnormality or an abnormal serum screening result. The detection of these abnormalities is in addition to those seen by conventional karyotyping, as all of the cases considered here were karyotypically normal by routine chromosome analysis.

Current conventional prenatal diagnosis requires an invasive procedure (amniocentesis or chorionic villus sampling) with an associated risk of miscarriage of approximately 0.5% to 1%.^27^ In many countries, the offer of an invasive procedure is usually preceded by maternal serum and/or ultrasound screening designed to stratify the risk primarily of Down syndrome in the index pregnancy. However, the increasing use of high resolution fetal ultrasound, particularly in the late second and early third trimesters, may reveal detailed abnormal phenotypes that may be associated with more subtle chromosome imbalances. Conventional prenatal cytogenetics has long been seen as the gold standard for chromosome diagnoses, but the resolution afforded by conventional G-banded chromosomes means that the vast majority of imbalances <6–10 Mb will go undetected.

From the results presented here, it is clear that the majority of studies to date have focused their ascertainment on cases with abnormal fetal ultrasound scans. This approach provides the highest rate of clinically relevant copy number changes in the presence of a normal conventional karyotype. However, although the overall pick-up rates in the non-ultrasound ascertainment groups is ∼1%, on a population level, this represents a large number of cases where clinically relevant copy number changes will go undetected if microarray technology is applied only to fetuses with an abnormal ultrasound. Furthermore, current prenatal diagnosis requires an invasive procedure with an ∼0.5% to 1% associated risk of miscarriage,^27^ and with the very low sensitivity of conventional cytogenetics to detect clinically significant chromosome abnormalities smaller than 6 to 10 Mb, it could be argued that once the invasive procedure has been carried out then microarray should be the frontline test once aneuploidies have been excluded by other methodologies (e.g. QF-PCR). The adoption of prenatal microarrays will also be significantly influenced by the method of healthcare delivery in any given country, especially whether such provision is publically or privately funded.

In this review, we have taken at face value the authors' categorisation of copy number changes especially those that have been placed within the ‘clinically relevant’ category. Antenatal diagnosis presents unique challenges especially when a clinician is confronted with a variant of unknown significance, and these challenges are often compounded by trying to match the antenatal phenotype with the genotype offered by the microarray results. In one of the large-scale studies reviewed here,^14^ genetic counselling was moderated by a review panel, which considered a number of the VOUS individually and came to a consensus as to whether it should, or should not, be reported back to the parents. Although in many instances a direct phenotype–genotype correlation can be made between an increasingly large number of pCNC and specific fetal phenotypes (e.g. a fetal outflow tract cardiac abnormality and a 22q11.2 DiGeorge deletion), serious consideration needs to be given to how copy number changes with known reduced penetrance and possible neurological involvement (e.g. 15q11.2 BP1–BP2 deletions) should be interpreted when detected antenatally. One potential source of assistance with such interpretation may be provided by penetrance estimates obtained by using Bayesian analysis models to compare the copy number variation frequencies in both control and clinically affected postnatal cohorts.^28^ A clear strategy will also be required for the handling of ‘incidental findings’, for example an X-linked deletion inherited from a phenotypically normal carrier mother when ascertained in a female fetus.

It should also be stressed that access to regularly updated international databases specifically designed to help facilitate genotype–phenotype correlations for copy number changes, neurodevelopmental phenotypes and congenital abnormalities in the postnatal setting will provide essential interpretative tools in the prenatal application of microarrays. Sites such as DECIPHER (http://decipher.sanger.ac.uk/), the International Standards for Cytogenomic Arrays (https://www.iscaconsortium.org/) and also multiple links via the major genome web browsers notably Ensembl (http://www.ensembl.org/Homo_sapiens/Info/Index) and the University of California Santa Cruz (http://genome.ucsc.edu/cgi-bin/hgGateway) all have updated relevant information concerning genotype–phenotype correlations. In the prenatal setting, it will also be crucial that expert interpretation of VOUS is monitored by experts in both Clinical Genetics and Molecular Cytogenetics before reports are issued to patients and their clinicians.

Rapid technological advances, especially the use of massively parallel sequencing to analyse cell-free DNA circulating in maternal plasma, raise the possibility that prenatal microarrays may have a limited time frame for clinical implementation. There are already a number of proof-of-principle studies that have demonstrated the feasibility of using non-invasive next generation sequencing approaches to detect not only the common aneuploidies but also unbalanced chromosome abnormalities including micro-deletions and micro-duplications.^29^–^32^ The pace at which these technologies will be applied to non-invasive prenatal testing will increase exponentially over the coming months providing significant challenges to all healthcare providers for assessing the most effective way of implementing these novel approaches. In any event, it is clear that the current model of screening followed by an invasive test and conventional karyotyping will soon be replaced by molecular approaches.

## CONCLUSION

Despite the perceived difficulties of implementing prenatal microarrays diagnostically, especially those associated with the discovery of VOUS and rarer incidental findings, the headline figures presented here indicate that microarray technology could and indeed should be the frontline prenatal test in the presence of a fetal structural abnormality. Furthermore, we argue that with a pick-up rate of at least 1% greater than that being achieved by karyotyping in the other ascertainment groups, and with the ever increasing availability of data to help with copy number change interpretation, the data presented here also provide support to the notion that microarrays should be the frontline test for all prenatal diagnoses regardless of ascertainment category.

**WHAT'S ALREADY KNOWN ABOUT THIS TOPIC?**

- Microarray testing gives an increase in detection rate of unbalanced structural abnormalities in both the postnatal and prenatal contexts.

**WHAT DOES THIS STUDY ADD?**

- This work attempts to calculate the overall detection rate, and the detection rates for different ascertainment categories, of clinically significant prenatal copy number changes detected by microarrays in the presence of a normal conventional karyotype by combining data obtained from several published studies.

## Supporting Information

Funding sources: Part of this work is awarded by the Efficacy and Mechanism Evaluation (EME) programme*, and is funded by the Medical Research Council (MRC) and managed by the National Institute for Health Research (NIHR) on behalf of the MRC-NIHR partnership. LSC is partially funded by the Great Ormond Street Hospital Children's Charity and the NIHR Biomedical Research Centre at Great Ormond Street Hospital. he views expressed in this publication are those of the author(s) and not necessarily those of the NHS, the NIHR or the Department of Health.

Supporting info item
